# Self-care practices and experiences of people living with HIV not receiving antiretroviral therapy in an urban community of Lusaka, Zambia: implications for HIV treatment programmes

**DOI:** 10.1186/1742-6405-10-12

**Published:** 2013-05-15

**Authors:** Maurice Musheke, Virginia Bond, Sonja Merten

**Affiliations:** 1 Zambia AIDS-related TB Research Project, University of Zambia, P.O Box 50697, Lusaka, Zambia; 2 Swiss Tropical and Public Health Institute, Socinstrasse 57, Basel CH-4002, Switzerland; 3 University of Basel, Faculty of Science, Basel CH-4003, Switzerland; 4 Department of Global Health and Development, London School of Hygiene and Tropical Medicine, Keppel Street, London WC1E 7HT, UK

**Keywords:** HIV, Self-care, Faith healing, Herbal remedies, Zambia

## Abstract

**Background:**

Despite the increasingly wider availability of antiretroviral therapy (ART), some people living with HIV (PLHIV) and eligible for treatment have opted to adopt self-care practices thereby risking early AIDS-related mortality.

**Methods:**

A qualitative study was conducted in urban Zambia to gain insights into PLHIV self-care practices and experiences and explore the implications for successful delivery of ART care. Between March 2010 and September 2011, in-depth interviews were conducted with PLHIV who had dropped out of treatment (n=25) and those that had opted not to initiate medication (n=37). Data was entered into and managed using Atlas ti, and analysed inductively using latent content analysis.

**Results:**

PHIV used therapeutic and physical health maintenance, psychological well-being and healthy lifestyle self-care practices to maintain physical health and mitigate HIV-related symptoms. Herbal remedies, faith healing and self-prescription of antibiotics and other conventional medicines to treat HIV-related ailments were used for therapeutic and physical health maintenance purposes. Psychological well-being self-care practices used were religiosity/spirituality and positive attitudes towards HIV infection. These practices were modulated by close social network relationships with other PLHIV, family members and peers, who acted as sources of emotional, material and financial support. Cessations of sexual relationships, adoption of safe sex to avoid re-infections and uptake of nutritional supplements were the commonly used risk reduction and healthy lifestyle practices respectively.

**Conclusions:**

While these self-care practices may promote physical and psychosocial well-being and mitigate AIDS-related symptoms, at least in the short term, they however undermine PLHIV access to ART care thereby putting PLHIV at risk of early AIDS-related mortality. The use of scientifically unproven herbal remedies raises health and safety concerns; faith healing may create fatalism and resignation with death while the reported self-prescription of antibiotics to treat HIV-related infections raises concerns about future development of microbial drug resistance amongst PLHIV. Collectively, these self-care practices undermine efforts to effectively abate the spread and burden of HIV and reduce AIDS-related mortality. Therefore, there is need for sensitization campaigns on the benefits of ART and the risks associated with widespread self-prescription of antibiotics and use of scientifically unproven herbal remedies.

## Background

While an estimated 5.9 million people in low and middle-income countries were on antiretroviral therapy (ART) by end of 2009, saving 14.4 million life-years since 1996 [1], this only represented half the estimated people eligible for treatment [2]. Previous studies indicate that patient retention in ART care is problematic. For instance, in sub-Saharan Africa (SSA), only an estimated 60% of patients were retained in ART programmes after 2 years of starting treatment [3,4]. This is despite the proven efficacy of ART in reducing AIDS-related deaths [5-8]. The reasons for patient attrition from ART care and failure to initiate treatment are varied and include financial costs associated with accessing treatment [9,10], fear of side effects [9,11-15], fear of drug toxicity and long-term harm to the body [14,15] and feeling healthy [11,14-17]. Other barriers are stigma [9,11], belief in faith healing [11,18-21], use of traditional medicine [17,18,20,22] and perceived burden of being on life-long treatment [12,14,23].

Zambia is one of the countries in SSA worst hit by the HIV pandemic, with an estimated HIV prevalence of 14.3% in the Zambian population aged 15–49 years [24]. The national HIV prevalence peaked at around 16% in the 1990s before levelling off and marginally declining to current rates [25]. Since 2005 when Zambia started providing free ART services in public sector health facilities, there has been a steady increase in the number of PLHIV accessing treatment, from 57,164 in 2005 to an estimated 344, 407 (adults and children) at the end of 2010, representing 68.4% of those in need of treatment [25]. Similar to studies elsewhere, barriers to accessing ART in Zambia include financial and logistical costs [26,27], concerns about side effects [15,27,28], negative perceptions of medication [15,26,28] and overcrowded health facilities [15,27]. Other barriers are stigma and fear of straining interpersonal relationships [27,28], fear of being on life-long treatment and inadequate/lack of food and nutritional support [28].

While the reasons for non-uptake of ART are known, there is paucity of evidence on how PLHIV eligible for but not on treatment manage their health condition; how PLHIV perceive and experience their health on account of any self-care practices; and the implications of self-care practices for successful delivery of ART care. Therefore, within a wider ethnographic study, this study explored the self-care practices and health experiences of PLHIV not receiving ART care in an urban community of Lusaka, Zambia. Self-care in this paper is defined as “the individual’s ability to manage the symptoms, treatment, physical and psychosocial consequences and lifestyle changes inherent in living with a chronic condition [in this case HIV] ability to monitor one’s condition and to effect the cognitive, behavioural and emotional responses necessary to maintain satisfactory quality of life” [29].

## Methods

### Research setting

The study was conducted in a predominantly low-income, high-density urban residential area of Lusaka, Zambia. Observations and interviews with study participants indicate that the area comprises multilingual ethnic groups with *Bemba* and *Nyanja* the most widely spoken local languages. Most residents are embedded in strong kin and non-kin social network relationships. Most of the families are large, in part due to the affect of HIV with some individuals growing up as orphans under the care of extended family members. Although not all family members lived together, they still maintained reciprocal social and economic support ties. Other social network relationships are a product of similar religious affiliations and occupational and social lifestyle activities. Due to lack of public social amenities, social lifestyle activities mostly revolve around patronising the ubiquitous bars and drinking places, often a prime source of sexual network relationships.

Most of the residents are poor. A few people are employed in low-paid jobs in the public and private sectors of the formal economy. The majority earned their living in the informal economic sector: as cross-border traders and local market traders mainly selling fruits, vegetables, meat products, charcoal and second-hand clothes either in the city centre markets or in the open-air local markets. Small makeshift shops, locally called *‘tuntemba’* are ubiquitous in the area - along the dusty and seasonally muddy roads and in the local open-air markets or next to homes. Unemployment is also endemic in the area especially among the lowly educated youth. The unemployment situation is further exacerbated by rural–urban migration as people move into the city in search of job opportunities and better life.

Health services are mainly provided by a public sector clinic which provides both in-patient and out-patient health services. The clinic also provides ART services. By mid-2010, the health centre had an estimated catchment population of over 150,000 with over 5,000 people on ART and over 5,000 registered for pre-ART care. Alongside the formal health system are traditional healers and herbalist who also provide ‘health’ care services, including for HIV. There is also a plethora of drug stores, some unlicensed, which provide over-the-counter medication.

Christianity is the dominant religion with a myriad of charismatic evangelical Pentecostal churches, some of which provide healing sessions for people with different ailments, including HIV.

### Research design and study participants

The paper draws on data from two exploratory qualitative lines of enquiry nested within a wider 18-month ethnographic study: namely patient attrition from ART care and barriers to patient initiation of ART. For the former, participants were PLHIV who had not reported for pharmacy pick up for at least 6 months hence being classified by the local ART clinic as lost-to-follow up (LTFU). The revised Zambian ART treatment guidelines have reduced the LTFU threshold to at least 60 days from the previous 180 days [30]. In the latter, participants comprised PLHIV recommended to start treatment by ART providers but declined. Only participants aged ≥18 years old constituted our study participants.

### Sampling strategy and recruitment of study participants

Purposive sampling using maximum variation sampling procedure was used to select study participants. The sampling technique allows for selection of participants with unique and diverse characteristics in order to generate in-depth unique insights and shared patterns that cut across cases. In this study, the diverse characteristics used to select study participants were demographic characteristics and length of time on and off HIV treatment. The sampling strategy facilitated collection of wide ranging, in-depth insights into self-care behavioural practices and experiences of PLHIV associated with not being on ART.

Due to the difficulties of identifying PLHIV who had dropped out of ART and those who had refused to initiate treatment, some PLHIV not on ART were first contacted and recruited through ART staff. They were contacted by mobile phone and/or physically followed up using locator information (physical address and mobile phone details) they had provided to ART providers at the time of enrolment for treatment. Other research participants were identified and recruited through community-based lay home-based care providers. Given that PLHIV had formed or belonged to social networks, a few study participants were recruited through snowball sampling in which PLHIV interviewed nominated and contacted on behalf of the researchers, friends and family members that they knew had discontinued HIV treatment or had opted not to start treatment despite it being recommended to them by ART providers. For both categories of PLHIV, only those that agreed to be interviewed were later contacted and followed up for interviews by the researchers.

### Data collection and analysis

Face-to-face, one-on-one, open ended in-depth interviews were conducted between March 2010 and September 2011. The main research question asked was: “how do you manage your HIV condition in the absence of ART?’ Follow-up interviews were conducted during the study period. The initial interviews usually lasted between 30–45 minutes and for those re-interviewed, follow-up interviews lasted between 15–20 minutes. All the interviews were recorded using a digital audio recorder. The majority of the interviews were conducted in the local language, *Nyanja,* and a few in English.

**Figure 1:**
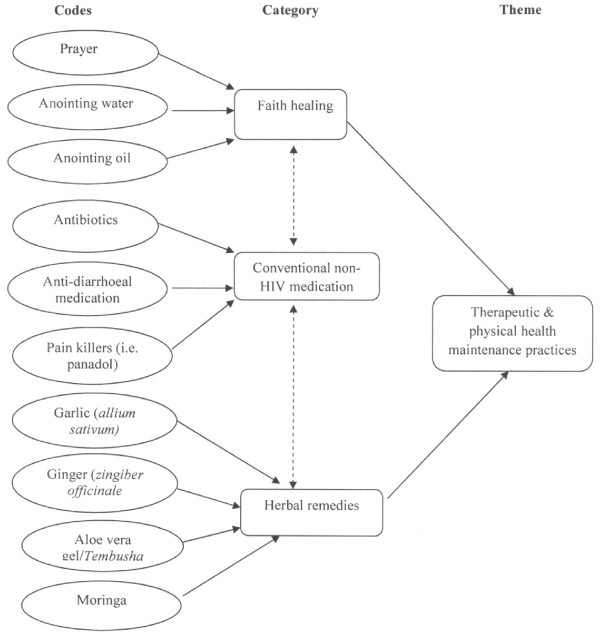
Example of coding framework of PLHIV self-care practices.

All interviews were transcribed verbatim. Data collection and preliminary data analysis was cyclical with some participants being re-interviewed and analysis continuously informing ensuing interviews. At the completion of data collection and transcription, all interview transcripts were entered into and organised and managed using Atlas ti version 6. Latent content analysis as described by Graneheim and Lundman [31] was undertaken. The transcripts were read several times to develop a sense of the whole data followed by open coding of the transcripts. The codes were compared for similarities and differences and then grouped into categories on a manifest level. Themes were generated by interpreting the categories for their underlying meaning (Figure 1). For instance, codes such prayer, anointing oil, anointing water were categorized as faith healing. Similarly, codes such as antibiotics, anti-diarrheal medication and pain killers were categorized as conventional non-HIV medication while garlic (*allium sativum*), ginger (*zingiber officinale*) aloe vera and *moringa* were categorized as herbal remedies. As a theme, the categories were collectively interpreted as therapeutic and physical health maintenance practices.

### Protection of research participants

Ethical approval was granted by the Ethics Committee of the State of Basel (Ethik-Kommission beider Basel) and the University of Zambia Humanities and Social Sciences Research Ethics Committee as part of a bigger research study, ‘Improving equity of access to HIV care and treatment in Zambia.’ The Ministry of health at national and district levels provided administrative clearance.

To ensure confidentiality and prevent involuntary disclosure of research participants’ HIV status, locations for interviews were based on the preferences of the respondents. Some interviews were conducted at the local public sector clinic and private spaces within the homes of respondents’ friends. A few PLHIV preferred being interviewed at home. This was done in the absence of family members to ensure privacy. Written informed consent was obtained from all research participants, including for quotes to be used anonymously in published reports.

## Results

A total of thirty-seven (37) PLHIV who declined to be enrolled into ART care and twenty-five (25) PLHIV who had abandoned ART care were interviewed. The majority of the respondents (n=41) were women and almost half (n=29) were aged between 25–34 years old. More than two-thirds (n=42) of the PLHIV were in informal employment. For the PLHIV who had stopped ART, eight (8) of the 25 PLHIV had been on treatment for less than a year; and less than half (n=10) abandoned medication in less than a year of being initiated on treatment. For PLHIV who had never been on ART, almost half (17 out of 37) were recommended ART within the year preceding the interviews (Table 1).

**Table 1:** Characteristics of PLHIV not on ART

**Characteristic**	**No. of PLHIV who dropped out of ART**	**No. of PLHIV never on ART**
**Age (Years)**		
18-24	5	4
25-34	11	18
35-44	3	14
>44	6	1
**Sex**		
Male	8	13
Female	17	24
**Marital status**		
Single	4	5
Married	12	22
Divorced/separated	7	6
Widowed		4
**Source of livelihood**		
Formal employment	4	5
Informal employment	15	27
Not working/dependant	6	5
**Time on treatment**		
<12 months	8	-
1-<2 years	6	-
2-<3 years	6	-
3-<4 years	2	-
≥4 years	3	-
**Duration not on treatment (up to time of interview)**		
6-<12 months	10	17
1-<2 years	8	10
2-<3 years	5	6
3-<4 years	2	4
4-<5 years	-	-
≥5 years	-	-

### Self-care practices of PLHIV not on ART

Seven (7) self-care practices were identified subsumed under four (4) main themes: Therapeutic and physical health maintenance; psychological well-being; healthy lifestyle and risk reduction practices.

### Therapeutic and physical health maintenance practices

#### Taking herbal therapies

Sustaining physical health involved taking herbal remedies to treat opportunistic infections and mitigate HIV symptoms, ‘wash toxins’ from the body, replace nutrients and improve appetite levels. Garlic (*allium sativum)*, ginger (*zingiber officinale*), aloe vera gel (also locally available in herbal plant form called *tembusha*), *moringa oleifera*, *Ngetwa* from Tanzania, crocodile fats, Chinese herbal remedies, *stametta* (aloe mixed with vitamins and herbs) and some herbs simply labeled ‘*Back to Eden’* were the most commonly used herbal remedies. Two PLHIV elucidated their experiences of herbal remedies:

*“I take some moringa. It is a tree with small leaves; we have it in the backyard of our house.... I just put it in tea or even in vegetables; we cook and mix it with other vegetables. Sometimes we “sashila” (add pounded groundnuts] to moringa and cook it as relish. Or you can even add the moringa to porridge.”* (50-year old woman; never been on ART)

*“You know with herbs, if you are coughing, you get guava leaves, lemon leaves, boil and drink them and then you are done. Even now, when my daughter is coughing, I would just take garlic, I mix with lemons, honey and make cough syrup and it is done.”* (42-year old woman; never been on ART)

For many PLHIV, herbal remedies were taken as a routine part of life regardless of whether they were experiencing illness-related symptoms or not. Some herbal remedies were cheap while others like aloe vera gel were expensive, fetching as high as US$30 per one litre container. Those with financial means were able to buy these herbal remedies from drug stores, pharmacies, herbal medicine traders and herbal medicine shops. Some PLHIV periodically relied on financial support of relatives to procure these herbal remedies. Some herbal remedies like *moringa* and *ginger*, comparatively cheap, were routinely added to food or tea or juice to treat or prevent illnesses such as cold and flu, diarrhoea or generally to allegedly ‘boost’ the immune system and reinvigorate the body.

Some PLHIV reported planting these ‘health-enhancing’ herbs, particularly *moringa* and *tembusha* at home to ensure easy and unfettered access. While ART required high levels of adherence, this was reportedly not the case with herbal remedies. PLHIV reported taking them if and when they wanted. The flexibility of herbal treatment requirements was in part the reason why some PLHIV found herbal remedies attractive compared to conventional HIV medication which required strict adherence. One woman proudly expressed her affection for these herbal remedies:

*“For me, I take tembusha and my CD4 cell count is high; that explains that the herbal remedy is working. So, why should I start ART?”* (32-year old woman; never been on ART)

One of the recurring themes was that while the study participants acknowledged the efficacy of ART, the burden of taking treatment for life, stringent adherence requirements and concerns about treatment side effects attracted them to use herbal remedies. One woman said:

*“Even if the ARVs work, people say they are for life and that they have side effect. So when people hear that, they decide to go for herbs because no one wants to experience the side effects or to take the medication for life. They say that herbs have no side effects.*” (23-year old woman; never been on ART)

#### Faith healing as HIV cure

Faith healing was also sought for curative purposes. Some PLHIV reported travelling outside Zambia in search of healing while others relied on their faith in God to secure healing:

“*When I went [to Nigeria] for healing, I was never the same person again…No one can deceive me that the power of God cannot work or does not work. I saw blind people gaining back their sight; I saw people in wheelchair walk; I saw people who could not hear start hearing. What more evidence does one need to believe that God performs miracles?* (48-year old man; stopped ART)

*“The thing is that when I pray, God tells me what to do. So God told me in a vision that you are healed and from the time that I prayed and stopped treatment, I have never been sick and have never taken any form of medication. The only secret which is there is to pray, and to believe in God, to have faith in Him.”* (45-year old man; stopped ART)

Ironically, some health workers with HIV, including those working in the ART clinic and eligible for ART also opted for faith healing and/or herbal remedies:

*“I have also taken anointing water from [....]. They say it cures if you have faith because it is prayed for by the man of God.”* (27-year old woman; Pharmacy Technologist)

#### Using conventional medicine, but only to treat HIV-related infections

Acutely aware of their fledgling health and limitations of other self care strategies, some PLHIV did not completely cut-off ties with the formal health system. In the absence of herbal medication or whenever symptoms did not abate, some PLHIV sought formal health care, but only to use conventional medicine to treat opportunistic infections. Skin infections, respiratory infections and gastrointestinal tract infections were the most commonly reported HIV-related ailments. In other instances, PLHIV got over-the-counter medication, from a plethora of drug outlets in the area and the city centre. Antibiotics such as septrin (co-trimoxazole prophylaxis), flagyl, amoxicillin, anti-diarrhoeal medication were the most reported conventional drugs used. Some PLHIV reportedly used antibiotics used by their spouses on ART. Two PLHIV narrated:

*“I just go to the clinic, they give me amoxil, and at times I go to the drug store and buy panadol or septrin or drugs for diarrhoea.... At the clinic, I just pretend as if I was never on ARVs because if I tell them, they [nurses] will just tell me to go to the ART clinic and get ARVs.”* (43 years old woman; stopped ART)

*“I take two tablets of septrin in the morning every day. When the drugs get finished, I go and buy…. Like today, I took my last drugs and I am planning to go and get some drugs from my friend on credit. She runs a drug store. She gives me on credit each time I do not have money to buy.*” (31-year old woman; stopped ART)

#### Periodic monitoring of CD4 cell count

Coupled with uptake of herbal remedies, faith healing and conventional non-HIV medication, some PLHIV also monitored their immune system through periodically seeking CD4 cell count testing. Those with the financial means went to private clinics while others made private arrangements with public sector clinic staff to access CD4 cell count testing. At private clinics, charges for CD4 count testing ranged between ZMK50, 000 (US$10) and ZMK 150,000 (US$30) per test. Financial support from family members enabled some PLHIV to meet the costs. One PLHIV explained this strategy:

*“I do my tests at a private clinic, there at Dr [.....]’s clinic. He tests for CD4 count. The last time I tested my CD4 count, it was in 2009 but time has elapsed since I last did my CD4 count.”* (43-year old woman; never been on ART)

### Healthy lifestyle practices

#### Dietary improvements

Good nutrition was a recurring theme as a self-care practice. While many PLHIV found this hard to sustain due to fragile livelihoods characterised by lack of job opportunities and steady and ‘adequate’ income, this problem was attenuated by improvising local food menus, for instance adding pounded groundnuts to vegetables like rape, cabbage, pumpkin leaves, locally called *“kusashila”* to nourish their bodies and improve their health condition. Other PLHIV heavily relied on nutritional supplements, sometimes supplied by relatives outside the country – in Europe, South Africa and United States of America:

*“I usually depend on a food supplement called ‘Shake herbal life’. It comes from America. It is in powder form. When you want, you can add it to milk or you can add even fruit juice. It is like powdered milk. What I do is, in the morning, I just take a glass of water and put two full tea spoons of the same ‘Shake’ and then I mix it and then I drink. If I have money, instead of water, I put milk, I mix it with milk and then I drink. If I have money and I have bananas, I blend and add it to my water and milk and then I drink. Those are my ARVs.”* (38-year old woman; never been on ART)

### Risk reduction practices

#### Safe sex and cessation of sexual relationships

To avoid re-infections particularly through unprotected sex, some PLHIV, especially those not married - either widowed or divorced/separated - reported either practicing safe sex or ceasing sexual relationships altogether. Some PLHIV drew lessons from the experiences of other PLHIV who had reportedly continued to live ‘healthy’ and ‘normal’ lives without ART partly because they had reportedly avoided re-infections. Two PLHIV explained:

*“From the time my husband died, I have vowed to abstain from sex. This has helped me because I learnt it from my HIV positive sister who has not been on any form of treatment since 1995 but is still alive and healthy today. Her secret is good diet and no sex.”* (42-year old woman; never been on ART)

*“Then we developed a culture in the house to say that let us start using condoms. In the house, it is condomising now. Then I think sometimes God comes in, you know. I have been praying to ensure that he would be agreeing to it, you know and for sure, he was not like, no what, what, and so we have been on condoms like that. He has been comfortable with it and we have been living like that.”* (38-year old woman; never been on ART)

### Psychological well-being self-care practices

#### “I rely on Jesus Christ”- religiosity and spirituality

Religiosity, particularly Christianity, was found to be woven into the day-to-day lives of some PLHIV, thus becoming a powerful self-care practice. Despite anticipation of stigma in religious circles due to the perceived association of HIV infection with improper sexual behaviour and a sinful lifestyle, through self-identification as Christians, some PLHIV, mostly women, drew on the biblical assurances of healing as a source of comfort and hope. Through organized religion, some PLHIV belonged to Christian denominations that were more accepting to PLHIV, particularly churches whose leaders or pastors professed to offer healing for people with HIV. While hoping for healing, some PLHIV reportedly put ‘everything in the hands of God’. They reported conducting private individual prayers with their pastors, going for collective intercessory prayers in which they opened up to one another and religious leaders about their HIV condition. One PLHIV who disclosed her status to her pastor described her unflinching belief in God:

“*As for me, I rely on Jesus Christ…. I just told myself that it is better I face Jesus Christ, God himself since He is the one that gave me life, not a human being. In John 14:1 [Bible], Jesus says ‘I am the way, the truth, and the Life.’ You know if God wants to take my life now, He can do it, whether I am on ARVs or not. I have a lot of friends who were on ARVs but have since died. Why? It is because man cannot preserve life; only God can.”* (48 year old HIV positive woman; stopped ART)

Another PLHIV explained her prayer life:

*“Even if you take the medicine, if you do not look to God for healing, you cannot get cured. So everything only works if you also involve God. So I also pray to God for me to be cured. I go for prayers every morning. There are inter-denominational prayer meetings here in Chawama, there at Pentecostal holiness in Kuku compound. Prayers are held every morning, everyday, Monday to Saturday. The prayers start at 06 hours up to 7 hours*.” (21-year old young woman; stopped ART)

To complement their prayer lives, some PLHIV reported using ‘anointing water’ - water believed to have healing properties after being prayed for by a Pastor believed to have spiritual healing powers - in order to ward off illnesses. They bought it from their churches that sold it or ordered it from Nigeria where a renowned Pentecostal tele-evangelist was reported to have spiritual healing powers. It cost at least US$100 for a 100 mls bottle. At a time when ART was free in public sector clinics, some PLHIV still opted for faith healing despite the associated financial costs. One PLHIV who went to Nigeria for prayers and bought the ‘anointing water’ explained:

*“It is called anointing water. I have got it right with me in the car. What happens is that he [Pastor] does not heal you, you heal yourself. The belief that you have got yourself that God can heal you is what heals you. The belief that tomorrow I will walk, that tomorrow my immunity will rise, it will. It is you and God, and I believe it. Belief is not medicine, but it also heals. You do not just need medicine to get healed. You also need belief.”* (48-year old man; stopped ART)

#### ‘I avoid thinking about it too much’: positive attitudes towards HIV infection

Some PLHIV acknowledged the relationship between psychological well-being and their immune system. Therefore, developing and maintaining positive attitude towards their HIV condition was viewed as another pre-condition for improved physical well-being. This meant reconstructing their lives to attain a sense of normalcy. PLHIV avoided ‘thinking too much’ about HIV and treated HIV as any other infection: “*I just try by all means to ignore it; I just pretend as if it does not exist. I tend to live a normal life”,* said *a 2*3-year old man, who had never been on ART. *“I do exercises; and I keep my mind very free. I avoid thinking about it too much,*” said another 24-year old man who has never been on ART. This enabled them to treat HIV not as a fatal condition; rather as something that they could live with and manage while acknowledging episodic periods of good and poor health.

#### The role of social network relationships

The self-care practices were modulated by kin and non-kin social network relationships. PLHIV treatment seeking behaviour was influenced by relationships with friends/family members and some formal health care providers. For instance, limited disclosure of HIV status to close and trusted friends and family members as well as formation of or belonging to informal networks of PLHIV facilitated coping and self-management of HIV. The social support received could be delineated into two: illness-specific support such as provision of financial resources to access herbal remedies and over-the-counter conventional medication to mitigate the HIV-related symptoms and obtain nutritional supplements. This type of support was mainly provided by family members:

*“So me, I just use herbal remedy called ‘palibe kantu’ (there is no problem) which my mother organises for me from the farm in Mumbwa (another district). This herb grows and spreads on the ground like sweet potatoes. So you peel it and boil it and start drinking the water. So she brings that for me from time to time.”* (32-year old woman; never been on ART)

The second form of social support mainly provided through religious groupings and networks with other people living with HIV involved psychosocial support. In addition, PLHIV also provided informational support to each other on illness management including which herbal remedies to use for what symptoms or ailments. They also shared information about sources of herbal remedies. As one PLHIV explained:

*“You know, you come to know each other when you start treatment because in a day, they enroll may be 10 people which means these ten people will be coming to the clinic on the say day plus those that have already started treatment. So, even when you stop taking ARVs, you reach a stage where you know each other, support each other, and even encourage one another.”* (39-year old woman; stopped ART)

In some cases, driven in part by their own treatment and health beliefs, it appeared that some health workers encouraged PLHIV to use faith healing and herbal remedies either in combination with or to the exclusion of ART care. Asked how she reconciled her non-uptake of treatment and counselling PLHIV to start and adhere to ART, one lay ART support worker explained:

*“You know, me even when I am at the ART clinic, I also encourage those people to say that it is not only ARVs, even herbs, because sometimes you find that somebody is taking ARVs, but still more the CD4 count keeps on dropping. You see, I would also advise them that can you also try this, try herbs.”* (42-year old ART support worker; never been on ART)

### For better and for worse: PLHIV perceptions and experiences of self-care practices

Some PLHIV reported a rebound in their CD4 cell count or improved physical health due to the use of herbal remedies or after going through prayer sessions and therefore believed that it was possible to live a normal and healthy life if one used herbal medication and/or ‘surrendered’ his life to God for care. One lay health worker who has never been on ART explained her experience:

*“When they told me that ‘your CD4 count is low’, I just started using herbs like moringa, and after that, my CD4 count improved a bit up to 460 something.”* (32-year old Treatment support worker; never been on ART)

Another PLHIV - an ART support worker - who went to South Africa for prayers instead of seeking ART care also reported a rebound in her CD4 cell count. She recounted:

*“We were taught how to receive healing. We were learning about how to have faith, and how to receive healing. And when we [with the husband] came back from there, I was just ok. When I came back, I went to the clinic to check my CD4 count and it had been boosted from 165 to 365. So that is it.*” (37-year old woman; never been on ART)

For some PLHIV, a rebound in physical health was not associated with previous use of ART. Instead, they attributed ‘good’ health to the effectiveness of their non-ART related self-care practices. One PLHIV who had gone to Nigeria for healing prayers explained his experience:

*“When I went there, I was not walking. Other people were assisting me to walk. But when I came from there, I was walking. He never gave me any herbs; he never gave me any medicine. He just gave me water and prayer.”* (48-year old man; stopped ART)

Notwithstanding the perceived effectiveness of these self-care strategies, some PLHIV reported noticeable decline in their health and all PLHIV contemplated starting ART if all other efforts failed. For others, a decline in health status was attributed to either lifestyle behaviour deemed incompatible with their condition or due to psychosocial distress. These included beer drinking, smoking, challenges of maintaining a nutritious diet, pregnancy and psychological distress due to strained family relationships. As two PLHIV explained:

*“The only problem why I develop these health problems, I look sick, is that I started drinking again. After two years of stopping treatment, I was ok. But that habit of drinking came back….The only mistake I made was that I started excessive drinking. Again I realized that this beer will kill me. So I stopped. I do not drink beer anymore.”* (48-year old man; stopped ART)

*“I used to monitor my CD4 count, and it was high… until 2008 when I was pregnant that is when my CD4 count came down.... But I think my CD4 count is also low because most of the time, we are quarrelling with my husband, so I think my mind is often disturbed.”* (32-year old woman; never been on ART)

Despite reporting a decline in health status, some PLHIV still remained upbeat, viewing their poor health as temporal - a period which every individual, regardless of their HIV status went through - and not an indication that they needed to start treatment. HIV treatment was viewed as not a guarantor of good health and their lives. To them, the death of PLHIV despite being on ART was emblematic of the shortcomings of ART:

*“It happens in life. Sometimes you can have good days and sometimes you can have bad days. So, if my health is not good today, it does not mean that it will be like that forever. Things change; starting treatment does not guarantee that you will become healthy forever. What about those people who have died despite taking ARVs?”* (24-year old man; stopped ART)

## Discussion

**Figure 2:**
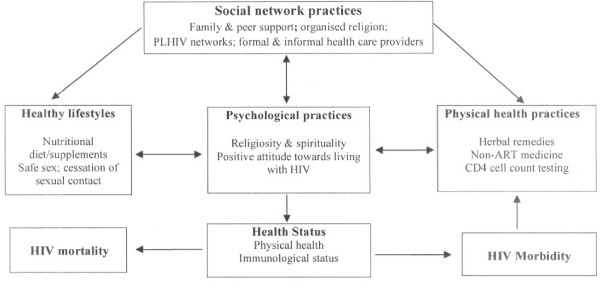
Nested relationships of self-care practices of PLHIV not on ART.

Our study explored the different self-care practices that PLHIV used as alternatives to ART care. We found that PLHIV used - often concurrently - herbal remedies, over-the-counter conventional medicine and faith healing, augmented by adoption of healthy lifestyle practices to promote physical and psychosocial well-being. If PLHIV opting for self care alternatives subsequently accessed health services, they never mentioned to non-ART prescribing health workers that they had opted to discontinue accessing ART or that they had opted not to start treatment. Neither did health workers inquire about the health and treatment history of these PLHIV. Thus, health workers missed the opportunity to bring these PLHIV back into ART care and treat them for the underlying cause of their ill-health. These self-care practices were modulated by availability of social support (Figure 2). These findings have implications for successful delivery of ART care.

### Herbal remedies may mitigation HIV symptoms but undermine access to treatment

Our findings show that herbal remedies were widely used as a self-care practice. The herbs were reportedly used to ‘cleanse’ the body of toxins, to treatment opportunistic infections, ‘boost’ the immune system and enhance physical functioning. The pressure associated with adhering to ART compared with herbal remedies that were taken on “if and when they want” basis motivated PLHIV to opt for herbal remedies. These herbs were perceived to be as effective as ART, albeit with perceived fewer side effects and no strict dosage and adherence requirements. Our findings are consistent with previous studies that have reported wide use of herbal medication as a self-care strategy [32-37].

Worryingly, the growth of the market for herbal remedies, their popularity and perceived effectiveness without scientific proof, risks undermining the success of ART care and consequently increasing the burden of HIV care in the long term. This could happen in three ways: first, by inhibiting patient (re-) entry into ART care and second, by ‘pulling’ out of ART care PLHIV already receiving medication due to the perception that herbal remedies were effective. Consequently, delayed initiation of ART may create additional therapeutic costs when PLHIV eventually return for ART care, albeit in poor health. The reported decline in health by some PLHIV clearly suggests that it is just a matter of time before PLHIV seek ART care. Third, the perceived effectiveness of herbal remedies could also attract even those already in ART care to use them. For these PLHIV, combining herbal remedies and antiretrovirals could affect the pharmacokinetics of antiretrovirals. This could either lead to higher antiretroviral plasma levels thus putting PLHIV at greater risk of developing serious side effects or lower the optimal therapeutic level of antiretrovirals thereby leading to treatment failure and an enhanced risk of developing drug resistance [38]. For instance, garlic, which is widely used as herbal therapy or nutritional supplement, has been reported to affect the pharmacokinetic concentration of HIV medication [39,40]. Bepe and colleagues [41] found that concomitantly using herbal remedies with antiretrovirals elevates some adverse events. Other studies have also reported reduced adherence to ART by those using herbal remedies and other alternative forms of care [42,43].

Importantly too, with growing demand for these herbal remedies and drawing on the desperation and naiveté of PLHIV, scientifically unproven herbal remedies could pose a health-risk to PLHIV. There have been reports about bogus HIV ‘cures’ or ‘immune boosters’ in Zambia and the dangers they presented to the lives and health of PLHIV. For instance in 2007, one of the purported AIDS ‘cure’ or ‘immune booster’ was found to be a pesticide, tetrasil, used for cleaning swimming pools [44]. Therefore, to ensure successful delivery of ART care, there is need for HIV care providers to step up sensitisation campaigns during ART counselling sessions and more widely about the health risks associated with use of unproven herbal remedies. This is critical not only to improve uptake of and adherence to treatment, avert possible negative interaction effects with antiretroviral treatment, but also to protect the health and lives of PLHIV and ensure patient retention in ART care.

### Religiosity and spirituality may simulate counselling but undermine uptake of ART care

Our study also found that some PLHIV sought recourse to religiosity and spirituality as a self-care practice. This is consistent with previous studies that have reported use of religiosity and spirituality as coping strategies by those with chronic health conditions, including HIV [45,46]. Religiosity has been associated with less psychological distress [47] and less depression [48], suggesting that it provides beneficial psychological outcomes. This is despite the reported stigmatizing influence of religious beliefs due to the association of HIV infection with improper sexual relationships [49,50].

In our study, religion and Christianity in particular was not only used to secure complete healing, but also to provide hope, comfort and assurances in the face of an incurable disease. While PLHIV were exposed to stigmatizing attitudes within religious settings, they often navigated their spiritual lives by moving to churches that preached healing and deliverance and whose leadership was perceived as open and receptive to PLHIV. Some PLHIV that were not publicly open about their HIV status only confided in and sought healing from their religious leaders. Two lessons can be drawn which have implications for successful delivery of ART care: On a positive note, these findings suggest that prayer simulates aspects of counselling [45] and can therefore be a useful coping mechanism for living with HIV. However, there is a thin line between faith healing as a psychosocial coping strategy and faith healing as a cure, with the latter posing a threat to patient access to and retention in ART care. More so, religious beliefs can trigger a fatalistic attitude and passive resignation with death, thus hindering uptake of treatment. Therefore, during group sensitisation and individual counselling sessions at ART clinics, health workers should tap into PLHIV belief systems to identify and harness those beliefs that promote psychosocial well-being without hindering PLHIV uptake of treatment. At community level, sensitisation efforts are required to encourage PLHIV claiming to have experienced healing through prayers to undergo formal medical tests to substantiate these claims. This is critical to ensure that PLHIV get (back) into ART care.

### PLHIV self-prescription of antibiotics raises concerns about development of microbial resistant strains

Due to the experiential limitations of faith healing and herbal remedies in addressing HIV-related ailments, we found that the use of conventional medicine, particularly antibiotics as a self-care strategy was common amongst PLHIV. Since the onset of ART roll-out, western medicines like co-trimoxazole prophylaxis have become widely available in low-income countries to treat microbial infections in PLHIV. The World Health Organisation (WHO) guidelines recommend provision of co-trimoxazole prophylaxis to all HIV symptomatic adults with CD4 count lower than 350 cells per μL in resource-limited settings [51]. Co-trimoxazole prophylaxis has been found to reduce mortality and morbidity amongst PLHIV through prevention and control of opportunistic infections [52,53]. Based on their knowledge of these conventional medicines and experiences with the formal health system and social network ties, PLHIV opted to obtain antibiotics in order to manage AIDS-related ailments. Our findings suggest that there was widespread use of antibiotics obtained either privately from the public sector clinics or purchased from a plethora of drug stores and pharmacies. The self-prescription of antibiotics raises great concern about future development of microbial resistance to antibiotics amongst PLHIV which could undermine future efforts to deal with HIV-related opportunistic infections. In the past, WHO has raised concerns about bacterial resistance to antibiotics due to their inappropriate use [54]. The reported widespread self-prescription and use of antibiotics by PLHIV therefore calls for sensitization measures about the dangers of self-prescription of antibiotics, especially amongst PLHIV. There is also need for enforcement of pharmaceutical regulations to control self-prescription and use of antibiotics, including clamping down on unregistered drug stores. This is even more critical for effective care of patients in ART care.

### Social support may also undermine uptake of ART care

As a corollary, self-care practices were found to be socially delineated and modulated by social support mechanisms. Social support “entails the structure of an individual’s social life (i.e. group memberships, existence of familial ties) and the explicit functions they serve such as emotional support” [55]. Previous studies have reported that social support was a strong predictor of utilization of formal HIV care services [56] and improved the odds of entry into and use of ART care [57]. Our findings suggest that while social support may serve as a stress-buffer and enables individuals through their social support networks to easily come to terms with their condition and develop a more positive outlook to life in the face of an incurable condition, social support is also a key self-care input factor that undermines access to ART care (Figure 2). For instance, social pressure and influence existed at family and interpersonal level to shun ART. Conversely, social support in the form of material, financial and moral support was provided to access herbal remedies, over-the-counter conventional medication to treat AIDS-related symptoms, and faith healing as cure for HIV. This places PLHIV at risk of AIDS-related morbidity and early mortality (Figure 2).

While the physical and psychological self-care practices complemented by adoption of healthy lifestyles and risk reduction such as practicing safe sex/cessation of sexual relationships to avoid re-infections are vital in the overall management of HIV, these behavioural practices in the absence of ART care are not sufficient to effectively prevent disease progression. The long-term benefits, therefore, reside in encouraging PLHIV to access ART care.

### Study limitations

Caution has to be taken when generalising the study findings. Some PLHIV were recruited in the community through their caregivers or snowball sampling and their eligibility for the study was based on self-reports of being eligible for treatment. Snowball sampling may have led to clustering of shared ideas and views. However, over two thirds of the study participants were identified, contacted and recruited through the ART clinic staff who were aware of which individuals had dropped out of ART or declined ART despite being eligible, suggesting that our sample largely comprised of treatment-eligible PLHIV who were independent from each other. Also, this study was descriptive and exploratory, aimed at providing wide ranging insights into how PLHIV managed their health in the absence of ART and how these self-care practices impacted uptake of ART. Thus, the purpose of the study was neither to establish causal relationships (i.e. effect of herbal remedies on immune system) nor to assess the effectiveness of these self-care strategies.

The paucity of data on PLHIV self-care practices meant that the study had to focus on identifying a wide range of themes rather than securing a representative sample of non-uptakers of treatment. To mitigate the potential for bias in the selection of study participants, we endeavoured to ensure that our study sample was diverse in terms of demographic, length of time since diagnosis and treatment-related characteristics. Therefore, similar studies are needed in other settings for comparability of findings. Despite these possible limitations, our findings could be generalised to similar settings in urban areas in the country and provide useful insight to inform policy and practice to improve ART uptake.

## Conclusions

This study explored the self-care practices of PLHIV not receiving ART. These PLHIV comprised those to whom ART was recommended by ART providers but for other reasons opted not to initiate treatment and PLHIV who had completely discontinued their medication. Our findings show that the use of herbal remedies and faith healing were dominant self-care practices. In addition, PLHIV used conventional medicines, but only to deal with AIDS-related ailments. It was also common for PLHIV to concomitantly use these different self-care practices. While these self-care practices were perceived by PLHIV to be effective, some PLHIV still reported slow and intermittent decline in health status, an indication that these self-care strategies were not as effective as reported and experienced. For PLHIV that had dropped out of ART, the perceived effectiveness of self-care practices could be attributed to previous use of antiretrovirals. These self-care practices undermine patient uptake of treatment and risk compromising future effectiveness of medical therapies and increase likelihood of microbial resistance and HIV resistant strains amongst PLHIV. In turn, this could increase the costs of ART care and family and community resources. Therefore, to ensure effective delivery of ART care, there is need to compile and include evidence of complementary HIV care practices into counselling guidelines and encourage those self-care practices that do not affect the effectiveness of antiretrovirals and do not put the health of PLHIV at risk. On the other hand, some of the self-care practices like self-prescription and indiscriminate use of antibiotics and use of scientifically unproven herbal remedies may cause long-term harm to PLHIV hence the need for sensitization campaigns and enforcement of pharmaceutical regulations to address them.

## Competing interests

The authors declare that they have no competing interests.

## Authors’ contributions

MM conceptualized the study, conducted data collection and analysis and wrote the draft manuscript. VB and SM contributed towards the conceptualization of the study, provided input in the analysis, interpretation of the findings and drafting of the manuscript. All authors have given final approval of the version to be published.
